# Mask Effectiveness for Preventing Secondary Cases of COVID-19, Johnson County, Iowa, USA

**DOI:** 10.3201/eid2801.211591

**Published:** 2022-01

**Authors:** Jacob Riley, Jamie M. Huntley, Jennifer A. Miller, Amelia L.B. Slaichert, Grant D. Brown

**Affiliations:** Johnson County Public Health, Iowa City, Iowa, USA (J. Riley, J.M. Huntley, J.A. Miller, A.L.B. Slaichert);; University of Iowa, Iowa City, Iowa, USA (G.D. Brown)

**Keywords:** 2019 novel coronavirus disease, coronavirus disease, COVID-19, severe acute respiratory syndrome coronavirus 2, SARS-CoV-2, viruses, respiratory infections, zoonoses, quarantine, public health, secondary attack rate, contact tracing, masks, Iowa, USA

## Abstract

In September of 2020, the Iowa Department of Public Health released guidance stating that persons exposed to someone with coronavirus disease (COVID-19) need not quarantine if the case-patient and the contact wore face masks at the time of exposure. This guidance differed from that issued by the Centers for Disease Control and Prevention. To determine the best action, we matched exposure information from COVID-19 case investigations with reported test results and calculated the secondary attack rates (SARs) after masked and unmasked exposures. Mask use by both parties reduced the SAR by half, from 25.6% to 12.5%. Longer exposure duration significantly increased SARs. Masks significantly reduced virus transmission when worn by both the case-patient and the contact, but SARs for each group were higher than anticipated. This finding suggests that quarantine after COVID-19 exposure is beneficial even if parties wore masks.

On September 29, 2020, the Iowa Department of Public Health (IDPH) issued new guidance for persons who had been in contact with someone infected with severe acute respiratory syndrome coronavirus 2 (SARS-CoV-2) (hereafter called case-patients). This guidance recommended that when both the case-patient and the contact were correctly and consistently masked during an exposure, the contact should perform symptom monitoring for 14 days instead of quarantining at home. This guidance deviated substantially from that provided by the Centers for Disease Control and Prevention (CDC), which still recommended at-home quarantine after exposure to someone with coronavirus disease (COVID-19), regardless of mask use. Johnson County Public Health (JCPH) staff decided to follow IDPH guidance but also supported any persons or organizations who chose to continue to follow the CDC recommendation.

Although the IDPH change in guidance provided an opportunity to lessen the burden of the pandemic on Johnson County, we were concerned about a potential increase in transmission rates. Because data supporting this change in guidance were lacking, we designed a prospective cohort study to evaluate the potential risk for increased virus transmission by measuring the secondary attack rates (SARs) of COVID-19 between persons exposed when both parties were masked and those exposed when >1 person was unmasked. 

The purpose of this study was to examine how effective masks are at reducing transmission of SARS-CoV-2 and, therefore, whether the new IDPH recommendation for symptom monitoring was appropriate. However, mask use is only 1 of many factors that affect SARS-CoV-2 transmission. While examining the available data, we identified several additional risk factors of interest, including symptom status, exposure setting, and exposure duration. This information enabled us to examine additional guidance relating to COVID-19, such as early release from quarantine and the potential for airborne transmission, to ensure that our recommendations did not increase the risk for transmission in the community. 

After reviewing relevant literature (*2*–*4*), we hypothesized that mask use consistent with CDC guidance (*5*) would reduce the SAR for COVID-19 in nonhousehold contacts from 10% to 5%. The study proposal was evaluated according to internal ethics review protocols, met the criteria for public health practice (*1*), and was not required to undergo institutional review board review.

## Methods

In March of 2020, IDPH issued a mandatory reporting order that required medical providers to report all COVID-19 test results and associated demographic information to IDPH each day. This information was then provided to each county-level public health department to enable case-patient investigation and contact tracing of residents who tested positive for COVID-19. We estimated that we would need a sample size of 1,200 contacts to detect a statistically significant difference in SARs between the 2 groups. We began collecting data on case-patients and their associated contacts that were reported to JCPH on or after October 20, 2020. By March 1, 2021, we had collected exposure and outcome information for ≈1,000 contacts and began to perform analyses while continuing to collect data for future calculations.

After being notified of new COVID-19 cases, we initiated contact with each person who tested positive for COVID-19. During this first contact, JCPH staff provided general isolation recommendations and obtained permission to send a link, via email or text message, to an online case investigation questionnaire that we had developed. This questionnaire collected basic information about demographics and households, details about symptoms, an overview of activities in the days before becoming ill, and a list of potentially exposed persons. The questionnaire was available in English, Spanish, and French. Case-patients also had the option to forgo the questionnaire and complete the investigation via phone interview, with the aid of a translation service if necessary.

After case-patients completed the questionnaire, we called each one to gather any additional information needed about their illness, to provide guidance for isolation and quarantine of household contacts, and to obtain a full list of close contacts. Close contacts were defined as persons who had been exposed to someone with a laboratory-confirmed case of COVID-19 during the case-patient’s infectious period within 6 feet for >15 minutes within a 24-hour period or who had experienced substantial direct exposure to a case-patient. Direct exposure is a somewhat subjective criterion and was evaluated on a case-by-case basis but could include exposures such as sharing food or drink, kissing, or shouting face to face in close proximity. In addition, on the basis of evidence of airborne transmission (*6*), JCPH classified persons as close contacts if they had spent >2 consecutive hours in the same enclosed space as a case-patient.

If the case-patient identified any close contacts during the case investigation, we asked for details about the exposure and contact: name, phone number, first and last date of exposure, whether the case-patient was masked, whether the contact was masked, if the case-patient was symptomatic at the time of exposure, exposure setting (indoors/outdoors/direct exposure), and exposure duration (>2 hours vs. <2 hours). We obtained this information from the case-patients because contacts were not provided with specific information about their exposure because of privacy concerns. This limitation also prevented us from collecting data about the type of mask worn by a close contact because the case-patient could not be expected to have this information and the contact would not know precisely when the exposure occurred. The many face coverings worn by Johnson County residents included 2-layer cloth masks, disposable surgical masks, double-layer gaiters, and KN95 masks. 

After obtaining a list of close contacts for a case-patient, JCPH staff called each identified close contact to gather additional information and provide appropriate quarantine recommendations. Information collected included additional demographic and contact information as well as information regarding the development of signs/symptoms, date of symptom onset, previous diagnosis of COVID-19, date of diagnostic test, COVID-19 vaccination history, and date(s) of vaccine administration. Contacts were also advised to undergo testing for COVID-19 during days 10–13 after their exposure or sooner if they experienced symptoms.

Throughout the study, we compiled data from the case-patient investigations and contact-tracing interviews into an internal database. We matched the data in our system to testing data from the state reporting system for each identified close contact. Contacts were included in the study if they met any of the criteria for a close contact; were exposed outside of a household, healthcare, or long-term care setting; investigators obtained data on mask use during the exposure for both the case-patient and the contact; and a laboratory-confirmed test result was collected 2–14 days after the date of exposure. We excluded from analysis persons who did not meet these criteria.

We computed SARs with 95% CIs for several individual risk factors, including combinations of masking status of case-patient and contact, exposure setting, whether the case-patient was symptomatic, and exposure duration. Subsequently, we conducted a multivariable logistic regression analysis by using these risk factors to ensure that the individual factors remained significant when combined. For the multivariable model, we combined case-patient and contact masking status into a score counting the number of persons masked (0, 1, or 2) because masking behaviors are highly correlated. Age was included as a numeric variable. Statistical significance is reported at a type 1 error rate of 0.05, and 95% CIs are reported.

## Results

**Figure 1:**
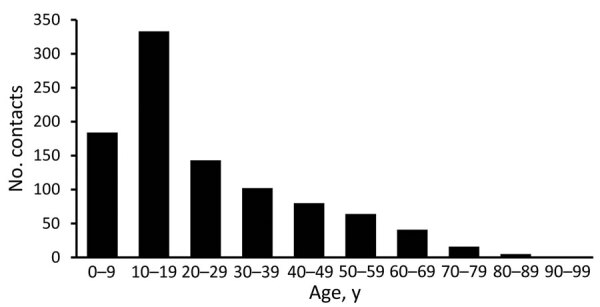
Age distribution of contacts in study of mask effectiveness for preventing secondary cases of coronavirus disease, Johnson County, Iowa, USA, October 23, 2020–February 28, 2021.

From October 23, 2020, through February 28, 2021, we identified 969 nonhousehold contacts who met inclusion criteria and for whom we were able to collect both exposure (mask usage) and outcome (test result) data. These 969 contacts were associated with 431 cases. The average number of contacts per case was 2.25 (range 1–13). Of these contacts, 3 had only an inconclusive test result and were not included in additional analyses. The age range of contacts was 0–90 years; median age was 18 years. The age distribution was skewed toward younger persons (0–18 years). Of the 966 contacts included in the analysis, 768 tested negative and 198 tested positive, resulting in an overall SAR of 20.5% (95% CI 18.1%–23.2%) (Figure 1).

To determine the effectiveness of masks for reducing SARS-CoV-2 transmission, we compared calculated SARs when both parties were wearing masks with SARs when >1 person was not wearing a mask at the time of exposure (Table 1). Most contacts (590, 61%) were exposed when >1 person was not wearing a mask. Of these 590 persons, 439 tested negative and 151 tested positive, leading to an SAR of 25.6% (95% CI 22.3%–29.4%). The remainder of the contacts (376, 39%) were exposed when both the case-patient and the contact wore masks during the exposure. Of these 376 persons, 329 tested negative and 47 tested positive, resulting in an SAR of 12.5% (95% CI 9.6%–16.3%). The sample sizes for subgroups of contacts exposed when >1 person was not masked were much smaller, but the SAR for contacts exposed when only the case-patient was masked was 29.1% (95% CI 19.3%–43.9%) and when only the contact was masked was 10% (95% CI 4.0%–25.3%).

**Table 1:** Mask effectiveness for preventing secondary cases of coronavirus disease, Johnson County, Iowa, USA

Mask use, case-patient/contact	Negative	Positive	Secondary attack rate (95% CI), %
Overall	768	198	20.5 (18.1– 23.2)
Total unmasked*	439	151	25.6 (22.3–29.4)
Unmasked/unmasked	364	131	26.4 (22.9– 30.7)
Unmasked/masked	36	4	10.0 (4.0– 25.3)
Masked/unmasked	39	16	29.1 (19.3–43.9)
Masked/masked	329	47	12.5 (9.6–16.3)
Unknown	69	23	25 (17.5–35.6)
School-age, 5–18 y			
Unmasked*	156	53	25.2 (20.1–32.0)
Masked/masked	191	26	12.0 (8.4–17.2)

*When >1 person was unmasked during exposure.

**Figure 2:**
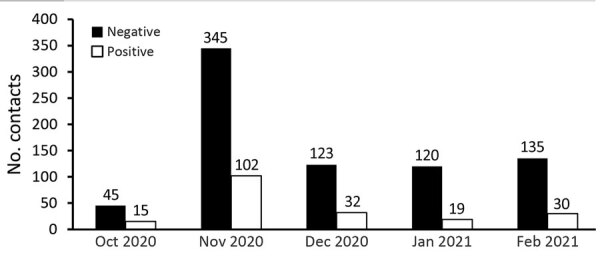
Number of contacts with test results during study of mask effectiveness for preventing secondary cases of coronavirus disease, Johnson County, Iowa, USA, October 23, 2020–February 28, 2021**.**

To ensure that our calculations were representative of the entire study period and not affected by specific outbreak or superspreader events, we examined the distribution of contacts over time (Figure 2). The number of cases in November increased, resulting in a corresponding increase in the number of contacts. The proportion of contacts testing negative compared with those testing positive remained roughly consistent throughout the study.

Because the age range of participants was skewed toward younger persons, we also calculated SARs for masked and unmasked exposures among school-age children (5–18 years of age) to ensure that our results were not affected by age distribution. Of the 966 contacts, 426 (44%) were within this age range. Of the 426 school-age children, 209 (49%) were exposed when >1 person was not masked; of those, 156 tested negative and 53 tested positive, resulting in an SAR of 25.2% (95% CI 20.1%–32.0%). A total of 217 (51%) school-age children were exposed when both persons were masked. Of those contacts, 191 tested negative and 26 tested positive, resulting in an SAR of 12% (95% CI 8.4%–17.2%). These results are consistent with our calculations for the entire study population.

To ensure that confounding was limited to the extent possible, we analyzed additional variables (Table 2). Overall SARs did not differ significantly when the contact was exposed while the case-patient was symptomatic (21.5%, 95% CI 18.1%–25.6%) compared with when the case-patient was not symptomatic (20.9%, 95% CI 17.4%–25.2%). In accordance with JCPH guidance, duration of exposure was measured as <2 hours or >2 consecutive hours. The SAR for exposures <2 hours was 13.5% (95% CI 9.6%–18.8%) and for exposures >2 hours was 25.6% (95% CI 22.2%–29.5%). SARs were lowest among those exposed while indoors (18%, 95% CI 15.1%–21.3%), followed by outdoors (25%, 95% CI 14.2%–44.0%), and highest among those who had been directly exposed (35.7%, 95% CI 17.7%–72.1%) (Table 2). Exposures for many contacts overlapped into multiple categories. The SAR for exposures that occurred in multiple settings was 25.8% (95% CI 18.4%–36.1%).

**Table 2:** Additional variables for study of mask effectiveness for preventing secondary cases of coronavirus disease, Johnson County, Iowa, USA

Variable	Negative	Positive	Secondary attack rate (95% CI), %
Case-patient			
Symptomatic	365	100	21.5 (18.1–25.6)
Not symptomatic	340	90	20.9 (17.4–25.2)
Exposure duration, h			
>2	413	142	25.6 (22.2–29.5)
<2	193	30	13.5 (9.6–18.8)
Exposure setting			
Indoors	488	107	18 (15.1–21.3)
Outdoors	27	9	25 (14.2–44.0)
Direct exposure	9	5	35.7 (17.7–72.1)
Multiple settings	72	25	25.8 (18.4–36.1)

**Figure 3:**
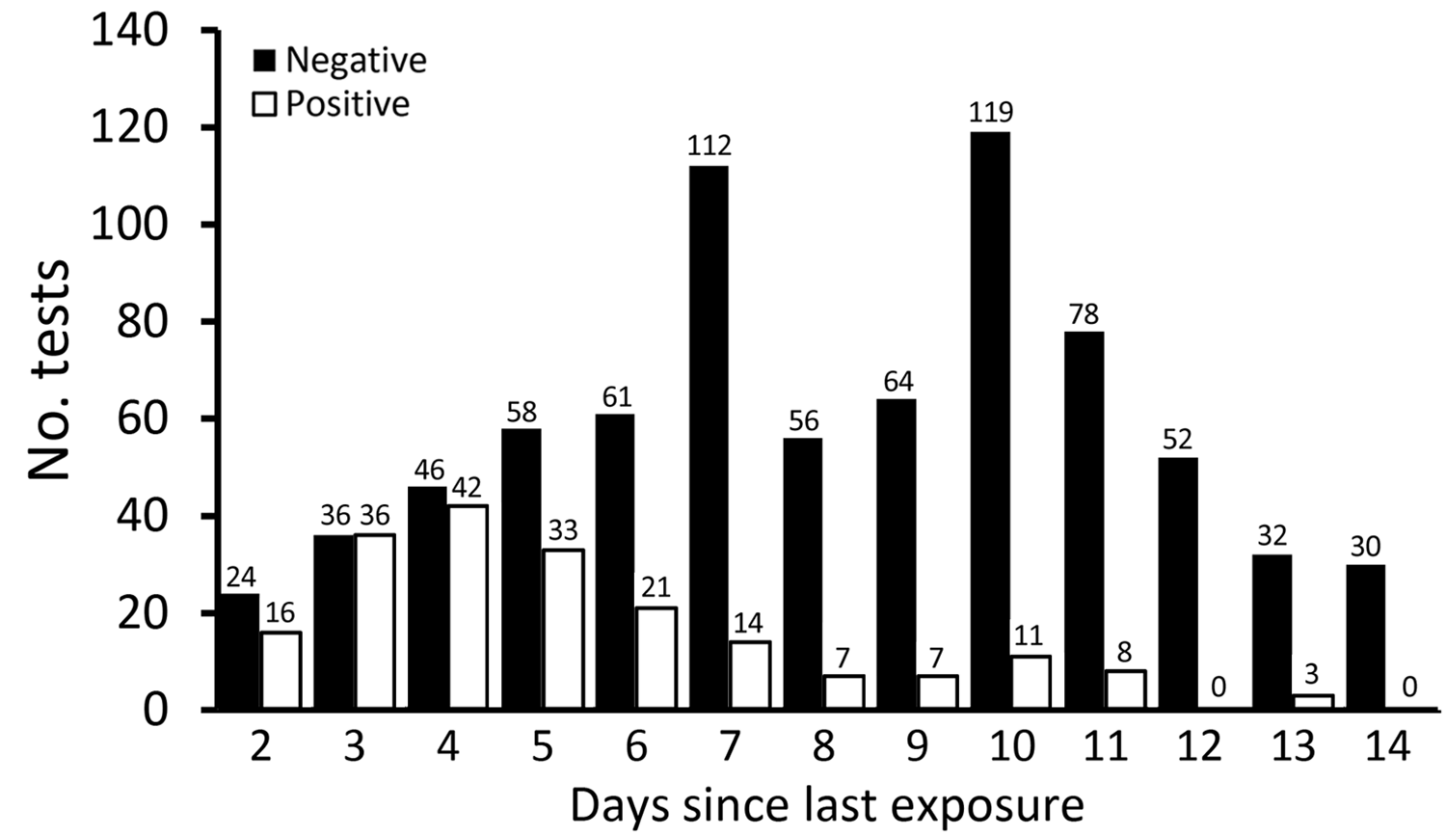
Days from exposure to coronavirus disease case-patient to testing of contact for disease, Johnson County, Iowa, USA, October 23, 2020–February 28, 2021.

On December 2, 2020, CDC issued guidance allowing early release from quarantine after 7 days with a negative test result collected 5–7 days after exposure or after 10 days without a test result for those who were asymptomatic (*7*). Because this guidance changed during our study period, we sought to examine the effects it might have on our results and transmission within our community. Our data collection methods enabled us to calculate the time from exposure to test and evaluate this guidance in the population of Johnson County. Of 198 contacts who tested positive, a total of 17 (8.6%) would have met criteria for early release and subsequently tested positive: 6 (3%) after 7 days on the basis of a negative test result and 11 (5.6%) after 10 days on the basis of absence of symptoms (Figure 3). This finding is consistent with the estimates provided by CDC guidance (*7*).

Other measured variables included vaccination before exposure and previous illness. All 16 contacts who reported >1 vaccination before exposure tested negative. Three contacts reported a previous positive test result; 2 had a previous positive test result within 90 days and tested negative after their exposure, whereas the remaining contact had a previous positive test result >180 days before exposure and again tested positive.

Several, but not all, risk factors of interest resulted in substantial differences in secondary SARs. To ensure that these factors remained significant in real-world settings, we performed a multivariable analysis. The multivariable results (Table 3) largely mirror the bivariate comparisons. Mask use was significantly associated with lower SARs (odds ratio [OR] 0.7, 95% CI 0.57–0.84); longer exposure was associated with higher SARs (OR 1.92, 95% CI 1.35–2.76); and age was positively associated with SAR (OR for 10-year increase 1.13, 95% CI 1.04–1.23). Indoor exposure was not significantly associated with SAR (OR 0.69, 95% CI 0.48–1.01), although it retained a negative nominal association. Variance inflation factors were examined, and the maximum value was 1.15, well below the problematic threshold for multicollinearity.

**Table 3:** Multiple logistic regression for study of mask effectiveness for preventing secondary cases of coronavirus disease, Johnson County, Iowa, USA

Parameter	Estimate	Odds ratio (95% CI)	p value
Intercept	−1.67	0.19 (0.11–0.32)	<0.001
Mask score	−0.36	0.70 (0.57–0.84)	<0.001
Exposure: indoors	−0.37	0.69 (0.48–1.01)	0.052
Case-patient symptomatic	0.25	1.28 (0.93–1.78)	0.131
Exposure >2 h	0.65	1.92 (1.35–2.76)	<0.001
Age, 0-y increase	0.13	1.13 (1.04–1.23)	0.003

## Discussion

Our goal with this study was to evaluate the change in quarantine guidance by examining the effectiveness of mask use for preventing transmission of SARS-CoV-2 and determining whether a resultant reduction in transmission was great enough to warrant symptom monitoring rather than quarantine of close contacts. The results from our analysis suggest that proper mask use is very effective for reducing transmission of SARS-CoV-2, lowering the SAR among contacts by half. However, consistent with a more recent study (*8*), SARs for both groups were notably higher than originally anticipated. On the basis of these findings, JCPH decided to recommend that persons follow CDC guidance after an exposure but also gave persons the option of following the less restrictive IDPH guidance.

Although sample sizes for subgroups of the unmasked cohort were relatively small, the evidence suggests that masks are more beneficial when worn by the contact than by the case-patient. This finding is further supported by the lack of a significant difference in SARs between contacts who had been exposed to an actively symptomatic case-patient compared with those exposed to a nonsymptomatic case-patient. However, specific symptoms were not included in this analysis. Transmission rates may be higher for persons experiencing symptoms such as cough or fever than for those experiencing symptoms such as headache and fatigue. In addition, we made no differentiation between asymptomatic and presymptomatic cases.

Duration of exposure was a significant predictor of SARS-CoV-2 transmission. JCPH recommends quarantine for persons exposed to a case-patient when indoors for >2 hours, regardless of distance, because of the potential for airborne transmission. Exposures lasting >2 hours were more than twice as likely to result in a positive test result than were exposures lasting <2 hours. We did not include distance as a measure in this study. We believed that distance would not be reliably self-reported and would probably not remain static for the duration of exposure, making any meaningful analysis challenging. Without measuring distance, it is impossible to quantify the number of contacts who were included in the study because their indoor exposure had been >2 hours but that had not been within 6 feet of the case-patient for >15 minutes. Despite this limitation, the difference in SARs between duration categories supports the assertion that airborne transmission occurs (*6*) because inclusion of any contacts exposed outside a 6-foot radius would otherwise decrease the difference in SARs between the 2 exposure-duration groups.

When we analyzed SAR by exposure setting, as expected the SAR was highest for contacts who had been directly exposed. Unexpectedly, the SAR was lower for persons who were exposed indoors than those who were exposed outdoors, although this finding did not remain significant in the multivariable analysis. This observed marginal association is potentially explained by several factors. Indoor exposures may have been more likely when persons were following social distancing recommendations. Outdoor exposures could have more often involved physical activities, resulting in higher respiration rates, or coincided with less adherence to social distancing.

Although our results suggest that mask use may not eliminate the need for quarantine, they indicate only a minor risk for increased transmission when adhering to shortened quarantine periods as outlined in CDC guidance (*7*). Only 17 contacts who tested positive would have met the criteria for early release, potentially infecting others. Most (79.5%) of the study population would benefit from a reduced quarantine without posing a risk to others. Because testing was not standardized among contacts, any predictive analysis would be unreliable, but the end result from this change in guidance is a significant reduction in burden to most contacts with only a slight increase in risk for transmission within the community.

Among the limitations to this study is that many persons could not be contacted or declined to cooperate with public health investigations. There are almost certainly substantial differences between case-patients and contacts that we were able to interview and those who declined to provide information or were unable to be reached. In addition, all of the data, with the exception of test results, were self-reported by either contacts or case-patients. Self-reported data can be unreliable. During investigations, case-patients may have had an incentive to provide false information to prevent friends, co-workers, or classmates from quarantining; or they may have demonstrated response bias by telling interviewers what they thought we wanted to hear. Although bias cannot be ruled out, we believe that persons who cooperate with public health investigations are more likely to provide accurate and honest information and to follow other public health guidance, such as social distancing and mask use. However, these challenges would bias our results toward the null, underestimating the benefit of mask use in the general population.

An additional limitation is related to generalizability. The population vaccination rate has risen dramatically since the period under study; we did not observe sufficient numbers of fully or partially vaccinated contacts to claim with certainty how masking policies would interact with changing population immunity. In addition, population immunity will be affected should any variants that escape the immune responses generated by >1 of the available vaccines emerge.

Last, although we detected several associations with SARs, the residual variability is substantial. When evaluated under 5-fold cross-validation, neither the logistic regression model nor a random forest version was able to produce predictions that were substantially above the no-information rate. This finding indicates that although we can quantify elevated risk, the measured information is not sufficient to predict transmission events on an individual level.

Nevertheless, we were able to measure a significant reduction in the rate of transmission when both persons were masked, which has useful implications for policy despite the continually shifting landscape of immunity and behavior. Despite the substantial reduction in transmission attributable to masking, the rate of transmission was still high when both parties were masked, leading us to conclude that in the absence of substantial hardship, quarantine regardless of mask use is generally preferred by public health practitioners. In reality, however, after less restrictive guidance has been issued, it is difficult to revert to recommendations that are more restrictive. This study highlights the value of creating public health guidance based on evidence rather than perception or public opinion.
